# 2165 Vesicular secretion of suppressor of cytokine signaling 3 by alveolar macrophages is dysregulated in NSCLC patients and its provision inhibits epithelial cell transformation and tumor cell function

**DOI:** 10.1017/cts.2018.148

**Published:** 2018-11-21

**Authors:** Jennifer Speth, Loka R. Penke, Joseph Bazzill, Douglas A. Arenberg, James J. Moon, Venkateshwar G. Keshamouni, Vibha N. Lama, Marc Peters-Golden

**Affiliations:** 1 University of Michigan School of Medicine; 2 Department of Internal Medicine, Division of Pulmonary and Critical Care Medicine, University of Michigan Health System, Ann Arbor, MI, USA; 3 Department of Pharmaceutical Sciences, University of Michigan College of Pharmacy, Ann Arbor, MI; 4 Graduate Program in Immunology, University of Michigan Medical School, Ann Arbor, MI

## Abstract

OBJECTIVES/SPECIFIC AIMS: Insufficient endogenous expression of suppressor of cytokine signaling 3 (SOCS3) with subsequent over-activation of its target, the transcription factor STAT3, has been associated with tumorigenesis and cancer development in the lung and other organs. We have observed that a “backup” source of SOCS3 in the lung, namely that secreted in microvesicles (MVs) by alveolar macrophages, is reduced in the bronchoalveolar lavage fluid (BALF) of KRAS mutant mice harboring lung tumors. Here we sought to evaluate levels of SOCS3 in BALF of a cohort of non-small cell lung cancer (NSCLC) patients and to test the effects of vesicular SOCS3 administration on tumor cell transformation and function as potential therapeutic strategy. METHODS/STUDY POPULATION: In total, 22 BALF samples were obtained from healthy volunteers (n=11) as well as patients undergoing diagnostic bronchoscopies for suspected lung cancer (n=11). SOCS3 levels in the BALF were determined by ELISA after brief sonication to disrupt vesicles. In vitro experiments utilized the human adenocarcinoma cell line (A549) or human G12V mutant KRAS-expressing rat lung epithelial cells (RLE-G12V). Proliferation, Fas ligand (FasL)-induced apoptosis, and chemical transformation with *N*-methyl-*N*′-nitro-*N*-nitrosoguanidine (MNNG) or cigarette smoke extract (CSE) were assessed by CyQuant assay, annexin V staining, and soft agar assay, respectively. For SOCS3 rescue, epithelial cells were treated with natural alveolar macrophages-derived MVs (isolated via ultracentrifugation) or synthetic unilamellar liposomes containing human recombinant SOCS3 for at least 1 hour before assay. RESULTS/ANTICIPATED RESULTS: SOCS3 levels were significantly reduced in BALF samples of patients determined to have NSCLC as compared with healthy volunteers (186.6±26.74 vs. 395.6±74.31 pg/mL, *p*=0.015, n=11). Addition of exogenous SOCS3-containing liposomes had the capacity to significantly inhibit MNNG and cigarette smoke extract-induced transformation and colony formation in soft agar. Exogenous SOCS3 provided in liposomes or in natural MVs significantly induced apoptosis (both in the presence and absence of FasL) and inhibited basal proliferation of A549 cells. DISCUSSION/SIGNIFICANCE OF IMPACT: These data identified a novel dysregulation of immune surveillance in the form of decreased SOCS3 secretion in the tumor-bearing lung that may contribute to tumorigenesis via sustained STAT3 activation. Future studies will focus on the mechanism underlying this defect and whether rescuing SOCS3 secretion can inhibit cancer progression in vivo.

